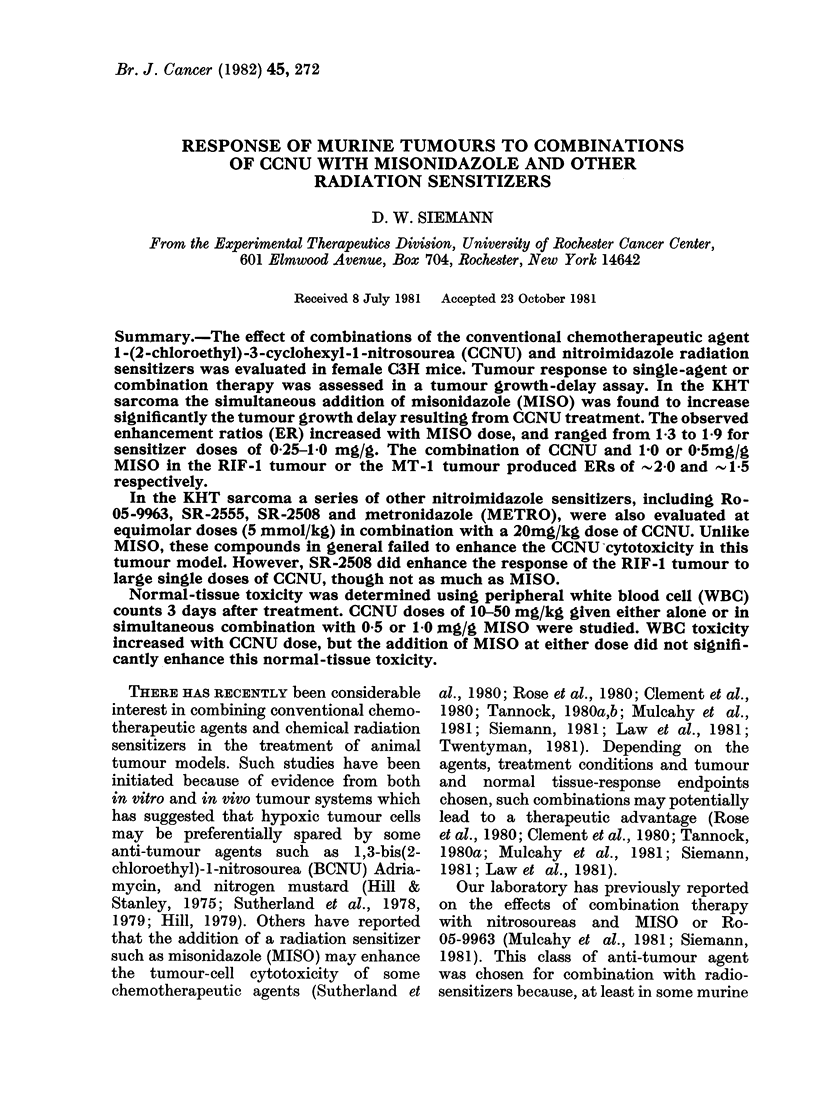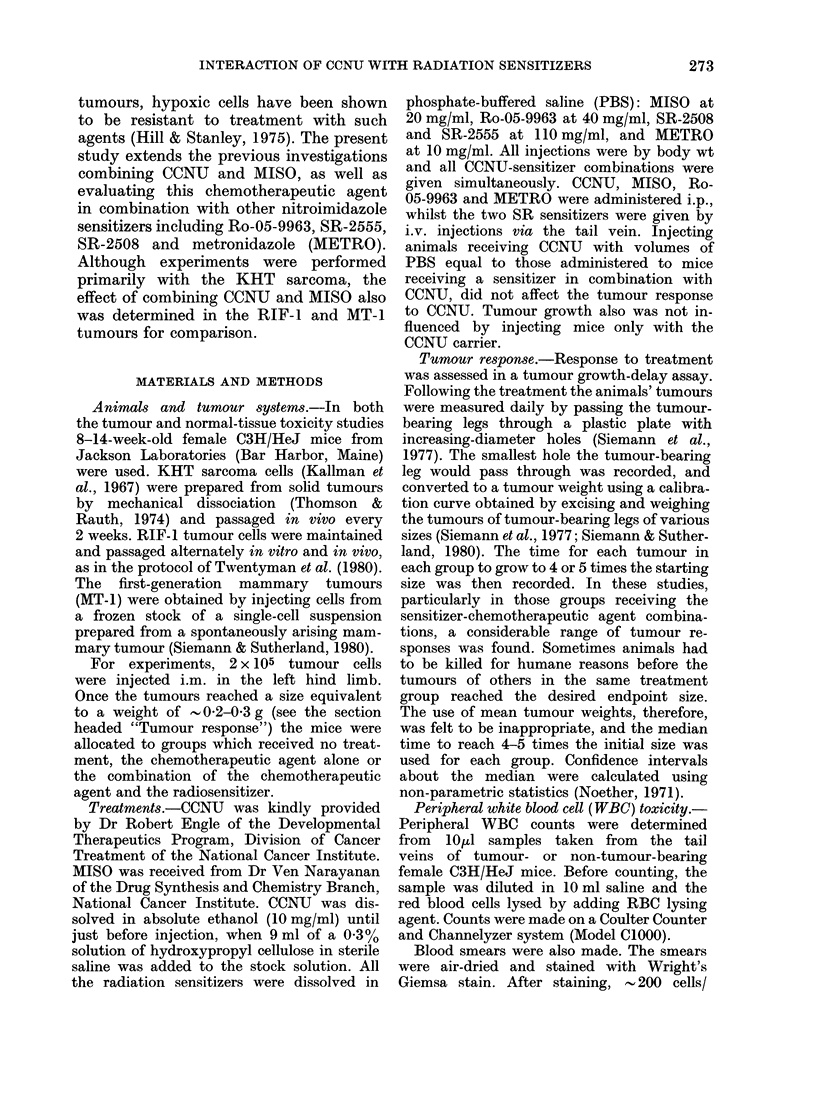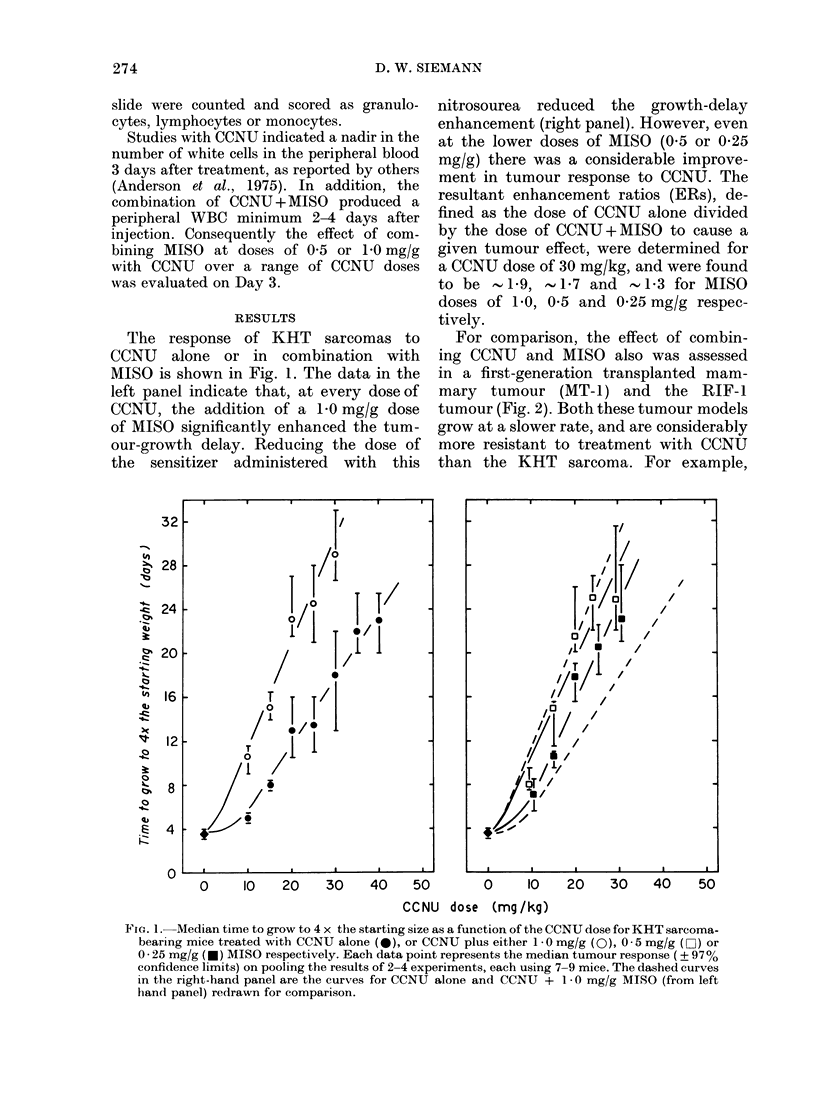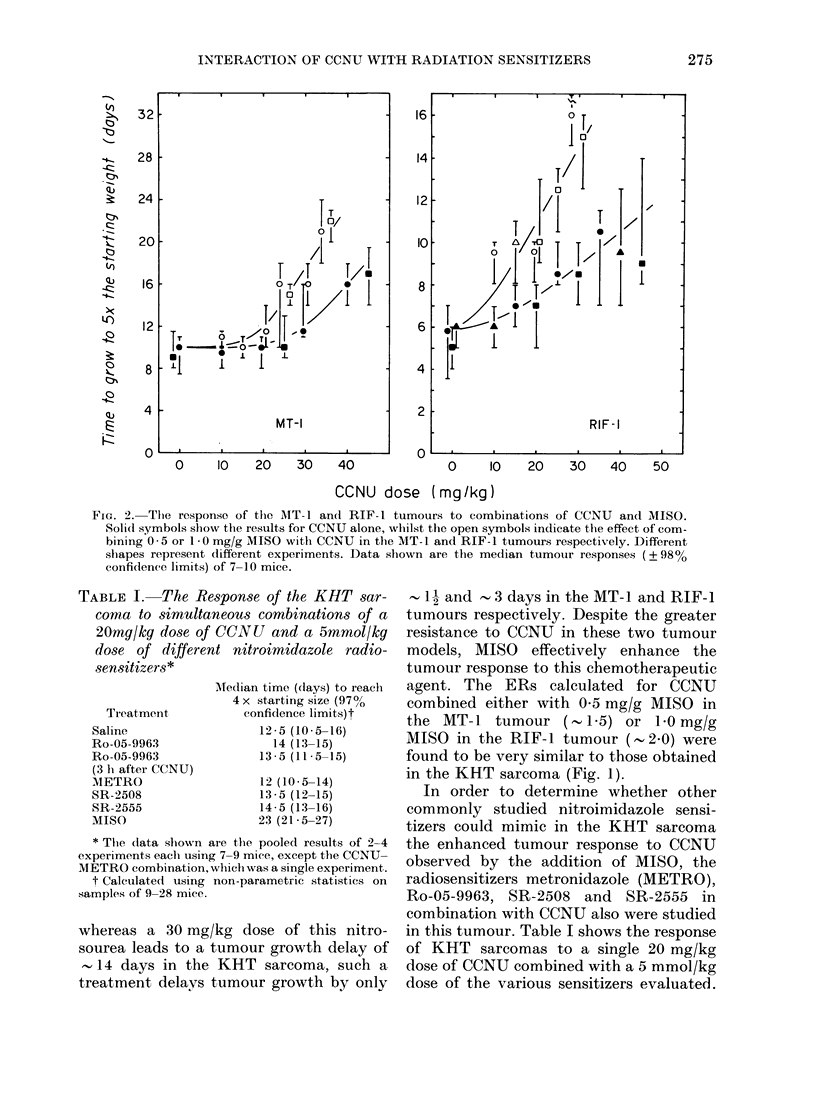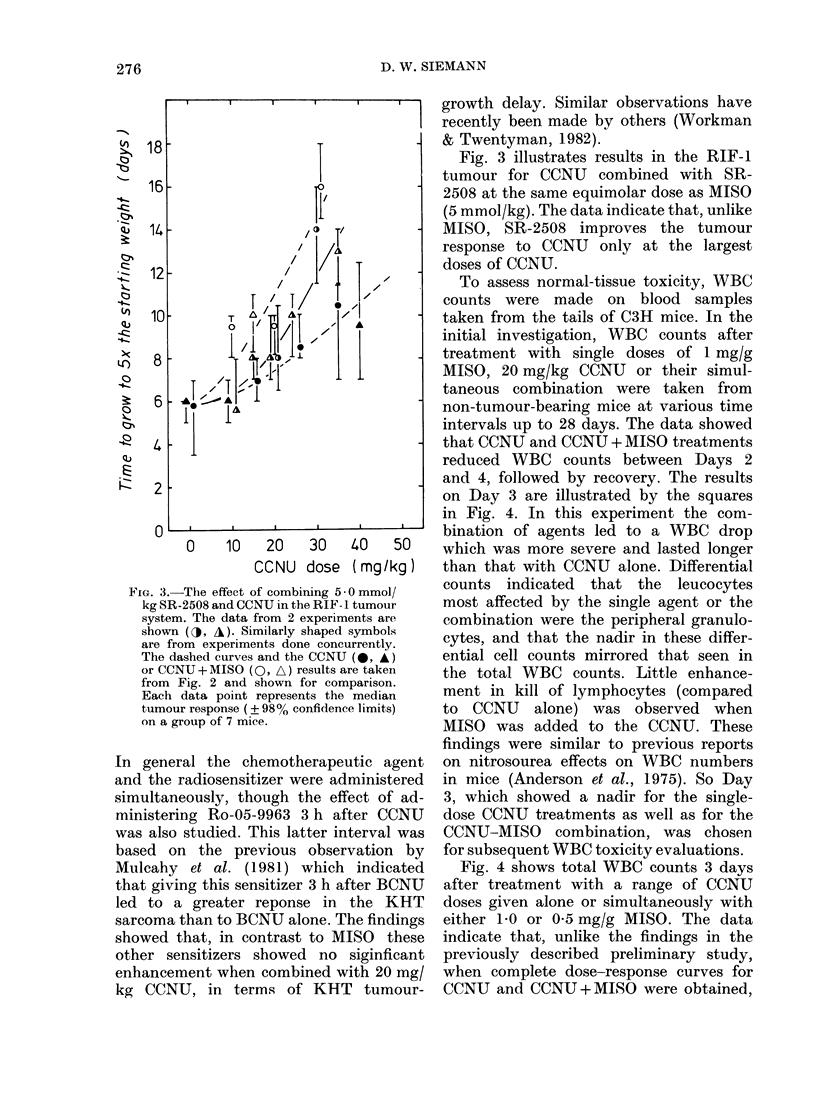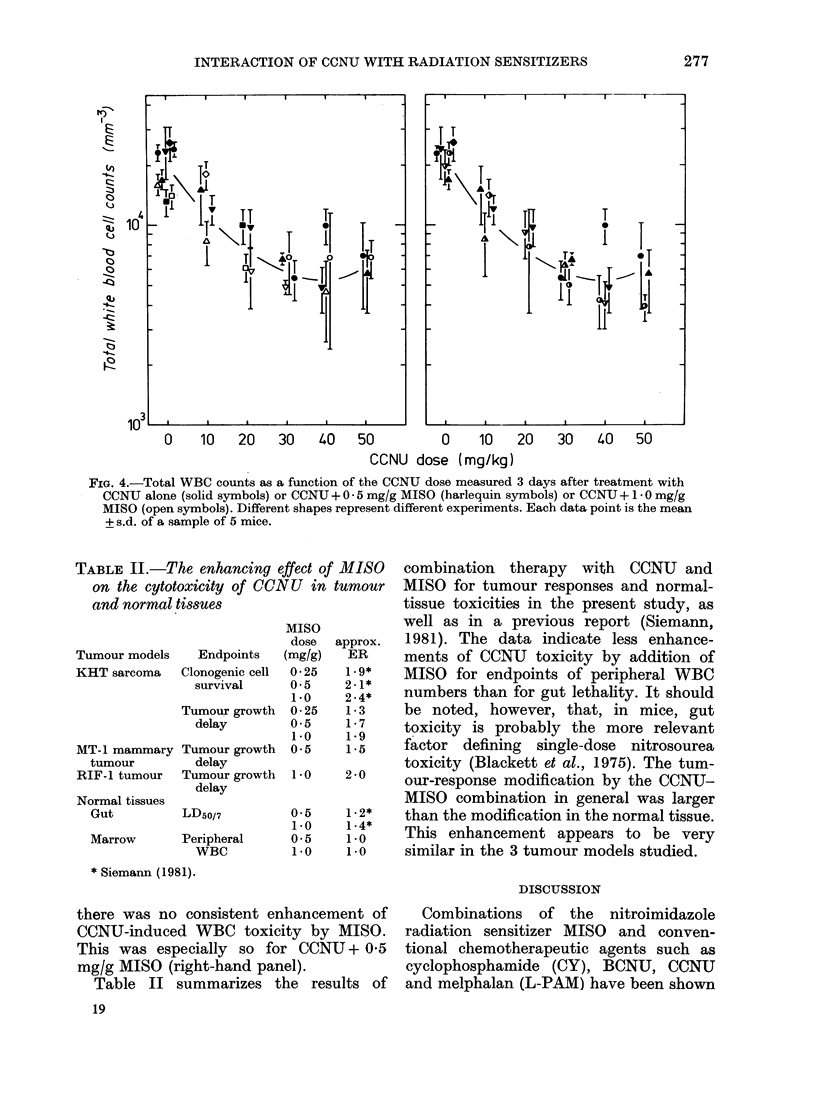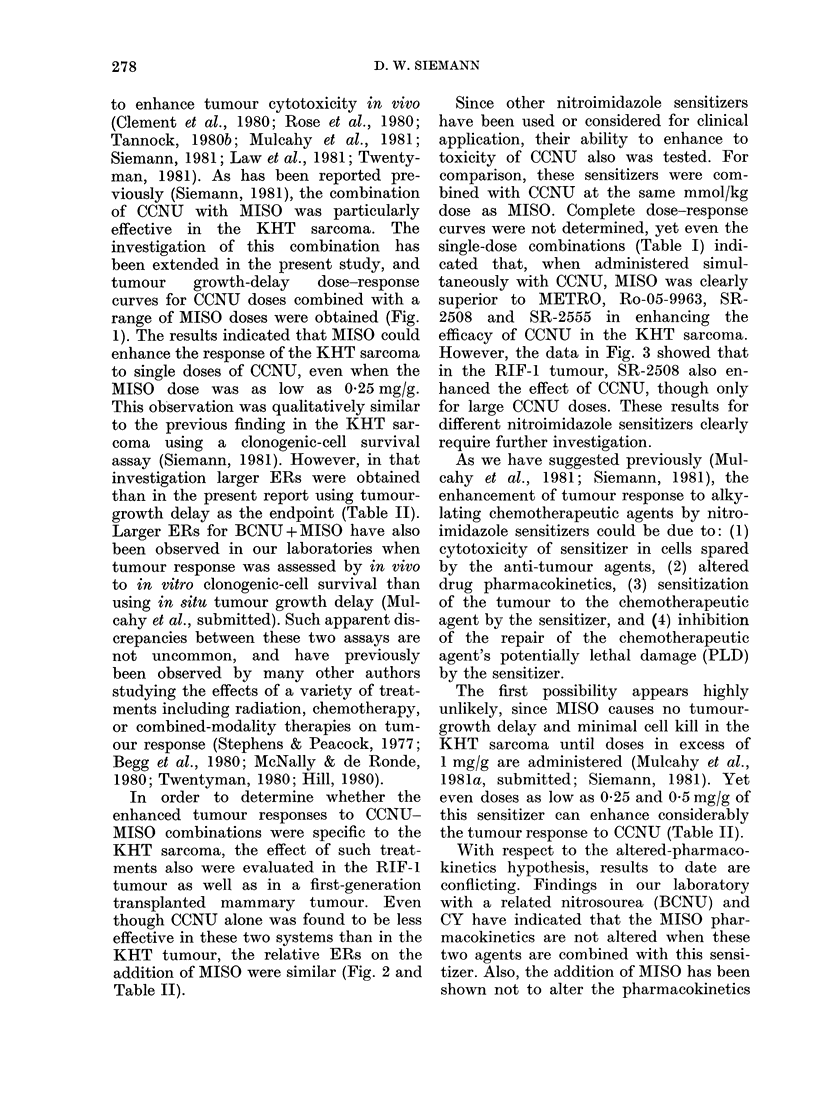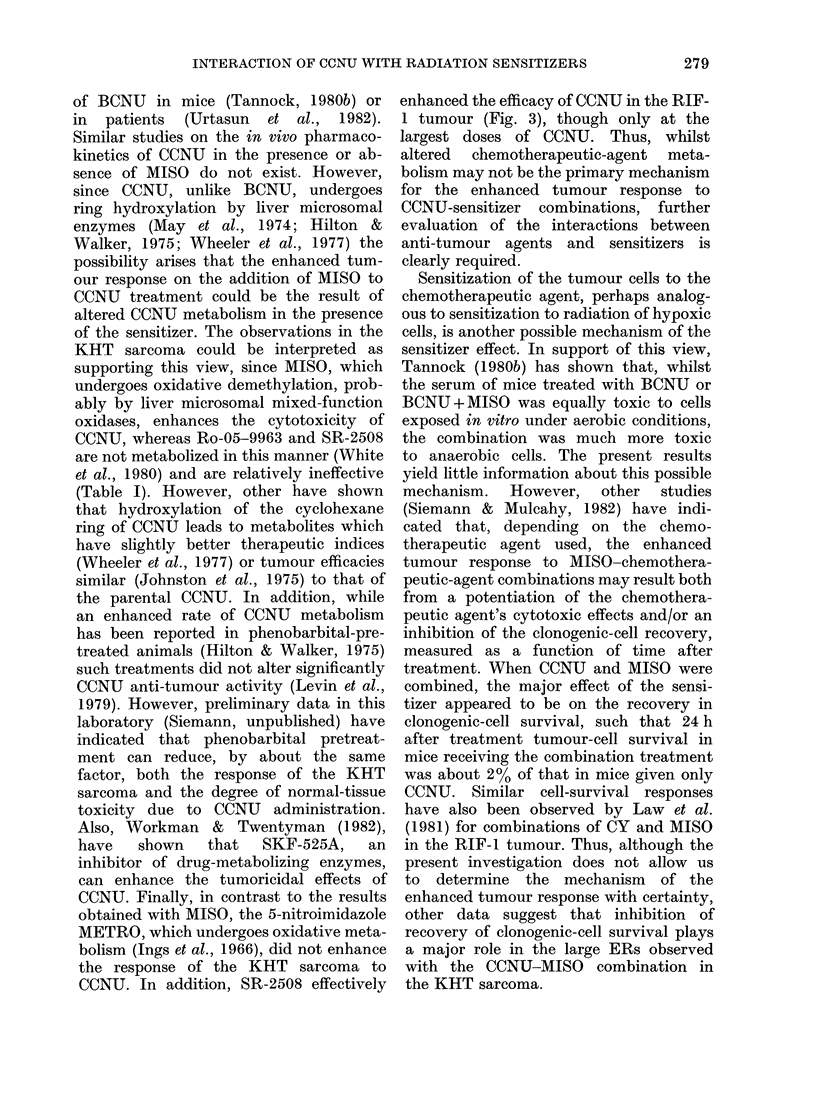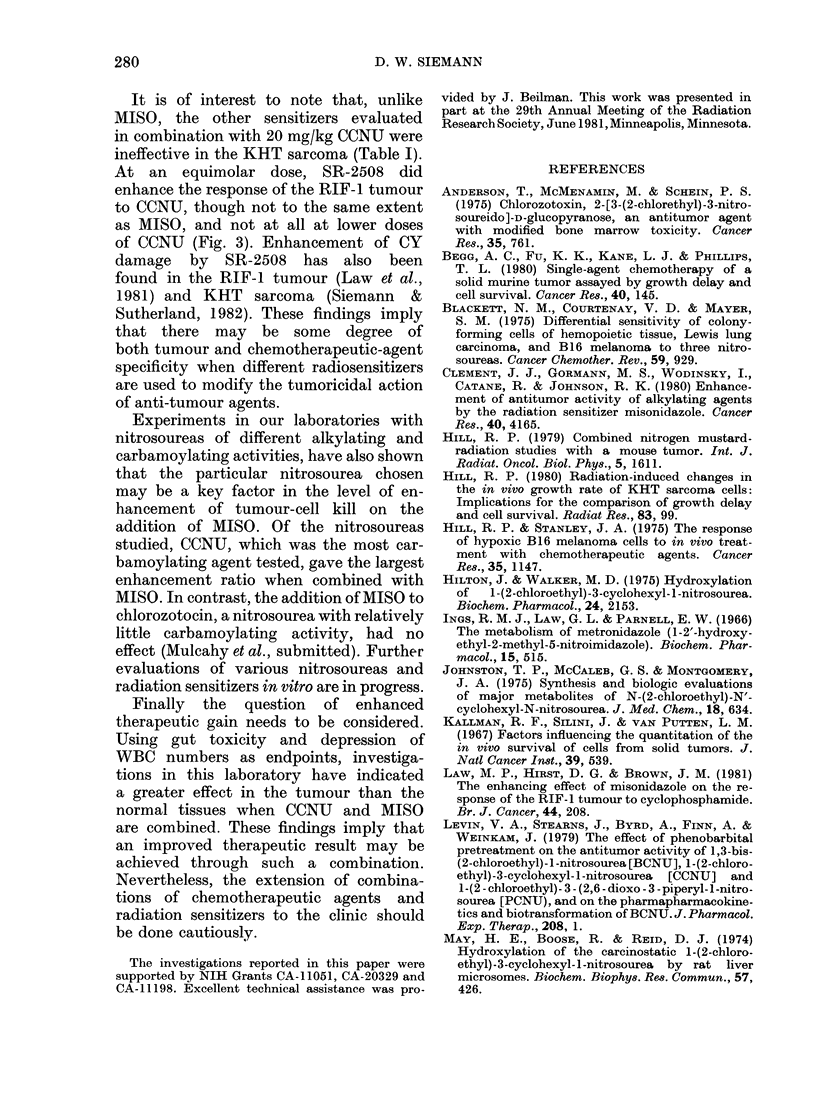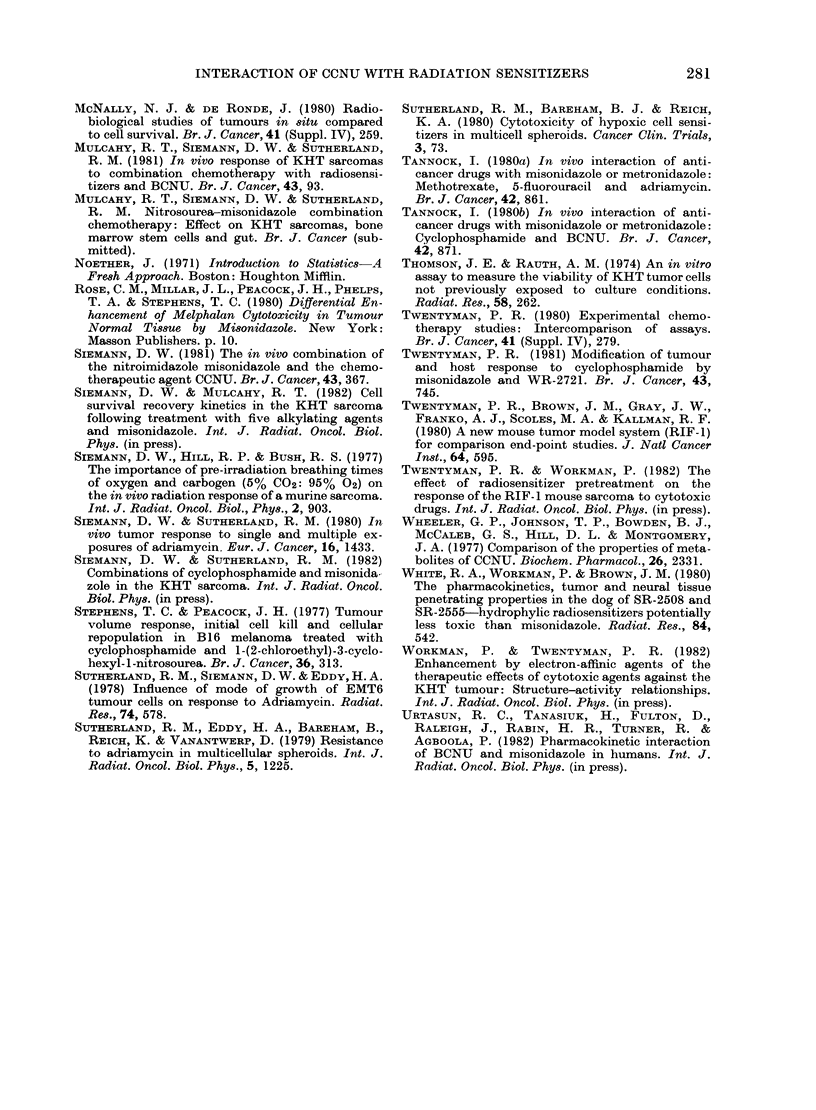# Response of murine tumours to combinations of CCNU with misonidazole and other radiation sensitizers.

**DOI:** 10.1038/bjc.1982.43

**Published:** 1982-02

**Authors:** D. W. Siemann

## Abstract

The effect of combinations of the conventional chemotherapeutic agent 1-(2-chloroethyl)-3-cyclohexyl-1-nitrosourea (CCNU) and nitroimidazole radiation sensitizers was evaluated in female C3H mice. Tumour response to single-agent or combination therapy was assessed in a tumour growth-delay assay. In the KHT sarcoma the simultaneous addition of misonidazole (MISO) was found to increase significantly the tumour growth delay resulting from CCNU treatment. The observed enhancement ratios (ER) increased with MISO dose, and ranged from 1.3 to 1.9 for sensitizer doses of 0.25-1.0 mg/g. The combination of CCNU and 1.0 or 0.5 mg/g MISO in the RIF-1 tumour or the MT-1 tumour produced ERs of approximately 2.0 and approximately 1.5 respectively. In the KHT sarcoma a series of other nitroimidazole sensitizers, including Ro-05-9963, SR-2555, SR-2508 and metronidazole (METRO), were also evaluated at equimolar doses (5 mmol/kg) in combination with a 20mg/kg dose of CCNU. Unlike MISO, these compounds in general failed to enhance the CCNU cytotoxicity in this tumour model. However, SR-2508 did enhance the response of the RIF-1 tumour to large single doses of CCNU, though not as much as MISO. Normal-tissue toxicity was determined using peripheral white blood cell (WBC) counts 3 days after treatment. CCNU doses of 10-50 mg/kg given either alone or in simultaneous combination with 0.5 or 1.0 mg/g MISO were studied. WBC toxicity increased with CCNU dose, but the addition of MISO at either dose did not significantly enhance this normal-tissue toxicity.


*Br. J. Cancer* (1982) **45**, 272

### RESPONSE OF MURINE TUMOURS TO COMBINATIONS OF CCNU WITH MISONIDAZOLE AND OTHER RADIATION SENSITIZERS

D. W. SIEMANN

*From the Experimental Therapeutics Division, University of Rochester Cancer Center, 601 Elmwood Avenue, Box 704, Rochester, New York 14642*

Received 8 July 1981 Accepted 23 October 1981

**Summary.—The effect of combinations of the conventional chemotherapeutic agent 1-(2-chloroethyl)-3-cyclohexyl-1-nitrosourea (CCNU) and nitroimidazole radiation sensitizers was evaluated in female C3H mice. Tumour response to single-agent or combination therapy was assessed in a tumour growth-delay assay. In the KHT sarcoma the simultaneous addition of misonidazole (MISO) was found to increase significantly the tumour growth delay resulting from CCNU treatment. The observed enhancement ratios (ER) increased with MISO dose, and ranged from 1·3 to 1·9 for sensitizer doses of 0·25–1·0 mg/g. The combination of CCNU and 1·0 or 0·5mg/g MISO in the RIF-1 tumour or the MT-1 tumour produced ERs of ~2·0 and ~1·5 respectively.**

**In the KHT sarcoma a series of other nitroimidazole sensitizers, including Ro-05-9963, SR-2555, SR-2508 and metronidazole (METRO), were also evaluated at equimolar doses (5 mmol/kg) in combination with a 20mg/kg dose of CCNU. Unlike MISO, these compounds in general failed to enhance the CCNU cytotoxicity in this tumour model. However, SR-2508 did enhance the response of the RIF-1 tumour to large single doses of CCNU, though not as much as MISO.**

**Normal-tissue toxicity was determined using peripheral white blood cell (WBC) counts 3 days after treatment. CCNU doses of 10–50 mg/kg given either alone or in simultaneous combination with 0·5 or 1·0 mg/g MISO were studied. WBC toxicity increased with CCNU dose, but the addition of MISO at either dose did not significantly enhance this normal-tissue toxicity.**

THERE HAS RECENTLY been considerable interest in combining conventional chemotherapeutic agents and chemical radiation sensitizers in the treatment of animal tumour models. Such studies have been initiated because of evidence from both *in vitro* and *in vivo* tumour systems which has suggested that hypoxic tumour cells may be preferentially spared by some anti-tumour agents such as 1,3-bis(2-chloroethyl)-1-nitrosourea (BCNU) Adriamycin, and nitrogen mustard (Hill & Stanley, 1975; Sutherland *et al.*, 1978, 1979; Hill, 1979). Others have reported that the addition of a radiation sensitizer such as misonidazole (MISO) may enhance the tumour-cell cytotoxicity of some chemotherapeutic agents (Sutherland *et al.*, 1980; Rose *et al.*, 1980; Clement *et al.*, 1980; Tannock, 1980*a,b*; Mulcahy *et al.*, 1981; Siemann, 1981; Law *et al.*, 1981; Twentyman, 1981). Depending on the agents, treatment conditions and tumour and normal tissue-response endpoints chosen, such combinations may potentially lead to a therapeutic advantage (Rose *et al.*, 1980; Clement *et al.*, 1980; Tannock, 1980*a*; Mulcahy *et al.*, 1981; Siemann, 1981; Law *et al.*, 1981).

Our laboratory has previously reported on the effects of combination therapy with nitrosoureas and MISO or Ro-05-9963 (Mulcahy *et al.*, 1981; Siemann, 1981). This class of anti-tumour agent was chosen for combination with radiosensitizers because, at least in some murine tumours, hypoxic cells have been shown to be resistant to treatment with such agents (Hill & Stanley, 1975). The present study extends the previous investigations combining CCNU and MISO, as well as evaluating this chemotherapeutic agent in combination with other nitroimidazole sensitizers including Ro-05-9963, SR-2555, SR-2508 and metronidazole (METRO). Although experiments were performed primarily with the KHT sarcoma, the effect of combining CCNU and MISO also was determined in the RIF-1 and MT-1 tumours for comparison.

### MATERIALS AND METHODS

*Animals and tumour systems.*—In both the tumour and normal-tissue toxicity studies 8–14-week-old female C3H/HeJ mice from Jackson Laboratories (Bar Harbor, Maine) were used. KHT sarcoma cells (Kallman *et al.*, 1967) were prepared from solid tumours by mechanical dissociation (Thomson & Rauth, 1974) and passaged *in vivo* every 2 weeks. RIF-1 tumour cells were maintained and passaged alternately *in vitro* and *in vivo*, as in the protocol of Twentyman *et al.* (1980). The first-generation mammary tumours (MT-1) were obtained by injecting cells from a frozen stock of a single-cell suspension prepared from a spontaneously arising mammary tumour (Siemann & Sutherland, 1980).

For experiments, $2 \times 10^5$ tumour cells were injected i.m. in the left hind limb. Once the tumours reached a size equivalent to a weight of $\sim 0{\cdot}2$–$0{\cdot}3$ g (see the section headed "Tumour response") the mice were allocated to groups which received no treatment, the chemotherapeutic agent alone or the combination of the chemotherapeutic agent and the radiosensitizer.

*Treatments.*—CCNU was kindly provided by Dr Robert Engle of the Developmental Therapeutics Program, Division of Cancer Treatment of the National Cancer Institute. MISO was received from Dr Ven Narayanan of the Drug Synthesis and Chemistry Branch, National Cancer Institute. CCNU was dissolved in absolute ethanol (10 mg/ml) until just before injection, when 9 ml of a 0·3% solution of hydroxypropyl cellulose in sterile saline was added to the stock solution. All the radiation sensitizers were dissolved in phosphate-buffered saline (PBS): MISO at 20 mg/ml, Ro-05-9963 at 40 mg/ml, SR-2508 and SR-2555 at 110 mg/ml, and METRO at 10 mg/ml. All injections were by body wt and all CCNU-sensitizer combinations were given simultaneously. CCNU, MISO, Ro-05-9963 and METRO were administered i.p., whilst the two SR sensitizers were given by i.v. injections *via* the tail vein. Injecting animals receiving CCNU with volumes of PBS equal to those administered to mice receiving a sensitizer in combination with CCNU, did not affect the tumour response to CCNU. Tumour growth also was not influenced by injecting mice only with the CCNU carrier.

*Tumour response.*—Response to treatment was assessed in a tumour growth-delay assay. Following the treatment the animals' tumours were measured daily by passing the tumour-bearing legs through a plastic plate with increasing-diameter holes (Siemann *et al.*, 1977). The smallest hole the tumour-bearing leg would pass through was recorded, and converted to a tumour weight using a calibration curve obtained by excising and weighing the tumours of tumour-bearing legs of various sizes (Siemann *et al.*, 1977; Siemann & Sutherland, 1980). The time for each tumour in each group to grow to 4 or 5 times the starting size was then recorded. In these studies, particularly in those groups receiving the sensitizer-chemotherapeutic agent combinations, a considerable range of tumour responses was found. Sometimes animals had to be killed for humane reasons before the tumours of others in the same treatment group reached the desired endpoint size. The use of mean tumour weights, therefore, was felt to be inappropriate, and the median time to reach 4–5 times the initial size was used for each group. Confidence intervals about the median were calculated using non-parametric statistics (Noether, 1971).

*Peripheral white blood cell (WBC) toxicity.*—Peripheral WBC counts were determined from 10 $\mu$l samples taken from the tail veins of tumour- or non-tumour-bearing female C3H/HeJ mice. Before counting, the sample was diluted in 10 ml saline and the red blood cells lysed by adding RBC lysing agent. Counts were made on a Coulter Counter and Channelyzer system (Model C1000).

Blood smears were also made. The smears were air-dried and stained with Wright's Giemsa stain. After staining, $\sim$200 cells/

slide were counted and scored as granulocytes, lymphocytes or monocytes.

Studies with CCNU indicated a nadir in the number of white cells in the peripheral blood 3 days after treatment, as reported by others (Anderson *et al.*, 1975). In addition, the combination of CCNU+MISO produced a peripheral WBC minimum 2–4 days after injection. Consequently the effect of combining MISO at doses of 0·5 or 1·0 mg/g with CCNU over a range of CCNU doses was evaluated on Day 3.

### RESULTS

The response of KHT sarcomas to CCNU alone or in combination with MISO is shown in Fig. 1. The data in the left panel indicate that, at every dose of CCNU, the addition of a 1·0 mg/g dose of MISO significantly enhanced the tumour-growth delay. Reducing the dose of the sensitizer administered with this nitrosourea reduced the growth-delay enhancement (right panel). However, even at the lower doses of MISO (0·5 or 0·25 mg/g) there was a considerable improvement in tumour response to CCNU. The resultant enhancement ratios (ERs), defined as the dose of CCNU alone divided by the dose of CCNU+MISO to cause a given tumour effect, were determined for a CCNU dose of 30 mg/kg, and were found to be ~1·9, ~1·7 and ~1·3 for MISO doses of 1·0, 0·5 and 0·25 mg/g respectively.

For comparison, the effect of combining CCNU and MISO also was assessed in a first-generation transplanted mammary tumour (MT-1) and the RIF-1 tumour (Fig. 2). Both these tumour models grow at a slower rate, and are considerably more resistant to treatment with CCNU than the KHT sarcoma. For example,

FIG. 1.—Median time to grow to 4 × the starting size as a function of the CCNU dose for KHT sarcoma-bearing mice treated with CCNU alone (●), or CCNU plus either 1·0 mg/g (○), 0·5 mg/g (□) or 0·25 mg/g (■) MISO respectively. Each data point represents the median tumour response (±97% confidence limits) on pooling the results of 2–4 experiments, each using 7–9 mice. The dashed curves in the right-hand panel are the curves for CCNU alone and CCNU + 1·0 mg/g MISO (from left hand panel) redrawn for comparison.

FIG. 2.—The response of the MT-1 and RIF-1 tumours to combinations of CCNU and MISO. Solid symbols show the results for CCNU alone, whilst the open symbols indicate the effect of combining 0·5 or 1·0 mg/g MISO with CCNU in the MT-1 and RIF-1 tumours respectively. Different shapes represent different experiments. Data shown are the median tumour responses (±98% confidence limits) of 7–10 mice.

TABLE I.—*The Response of the KHT sarcoma to simultaneous combinations of a 20mg/kg dose of CCNU and a 5mmol/kg dose of different nitroimidazole radiosensitizers**

| Treatment | Median time (days) to reach 4 × starting size (97% confidence limits)† |
|---|---|
| Saline | 12·5 (10·5–16) |
| Ro-05-9963 | 14 (13–15) |
| Ro-05-9963 (3 h after CCNU) | 13·5 (11·5–15) |
| METRO | 12 (10·5–14) |
| SR-2508 | 13·5 (12–15) |
| SR-2555 | 14·5 (13–16) |
| MISO | 23 (21·5–27) |

* The data shown are the pooled results of 2–4 experiments each using 7–9 mice, except the CCNU–METRO combination, which was a single experiment.

† Calculated using non-parametric statistics on samples of 9–28 mice.

whereas a 30 mg/kg dose of this nitrosourea leads to a tumour growth delay of ~14 days in the KHT sarcoma, such a treatment delays tumour growth by only ~$1\frac{1}{2}$ and ~3 days in the MT-1 and RIF-1 tumours respectively. Despite the greater resistance to CCNU in these two tumour models, MISO effectively enhance the tumour response to this chemotherapeutic agent. The ERs calculated for CCNU combined either with 0·5 mg/g MISO in the MT-1 tumour (~1·5) or 1·0 mg/g MISO in the RIF-1 tumour (~2·0) were found to be very similar to those obtained in the KHT sarcoma (Fig. 1).

In order to determine whether other commonly studied nitroimidazole sensitizers could mimic in the KHT sarcoma the enhanced tumour response to CCNU observed by the addition of MISO, the radiosensitizers metronidazole (METRO), Ro-05-9963, SR-2508 and SR-2555 in combination with CCNU also were studied in this tumour. Table I shows the response of KHT sarcomas to a single 20 mg/kg dose of CCNU combined with a 5 mmol/kg dose of the various sensitizers evaluated.

FIG. 3.—The effect of combining 5·0 mmol/kg SR-2508 and CCNU in the RIF-1 tumour system. The data from 2 experiments are shown (◑, ▲). Similarly shaped symbols are from experiments done concurrently. The dashed curves and the CCNU (●, ▲) or CCNU+MISO (○, △) results are taken from Fig. 2 and shown for comparison. Each data point represents the median tumour response (±98% confidence limits) on a group of 7 mice.

In general the chemotherapeutic agent and the radiosensitizer were administered simultaneously, though the effect of administering Ro-05-9963 3 h after CCNU was also studied. This latter interval was based on the previous observation by Mulcahy *et al.* (1981) which indicated that giving this sensitizer 3 h after BCNU led to a greater reponse in the KHT sarcoma than to BCNU alone. The findings showed that, in contrast to MISO these other sensitizers showed no siginficant enhancement when combined with 20 mg/kg CCNU, in terms of KHT tumour-growth delay. Similar observations have recently been made by others (Workman & Twentyman, 1982).

Fig. 3 illustrates results in the RIF-1 tumour for CCNU combined with SR-2508 at the same equimolar dose as MISO (5 mmol/kg). The data indicate that, unlike MISO, SR-2508 improves the tumour response to CCNU only at the largest doses of CCNU.

To assess normal-tissue toxicity, WBC counts were made on blood samples taken from the tails of C3H mice. In the initial investigation, WBC counts after treatment with single doses of 1 mg/g MISO, 20 mg/kg CCNU or their simultaneous combination were taken from non-tumour-bearing mice at various time intervals up to 28 days. The data showed that CCNU and CCNU+MISO treatments reduced WBC counts between Days 2 and 4, followed by recovery. The results on Day 3 are illustrated by the squares in Fig. 4. In this experiment the combination of agents led to a WBC drop which was more severe and lasted longer than that with CCNU alone. Differential counts indicated that the leucocytes most affected by the single agent or the combination were the peripheral granulocytes, and that the nadir in these differential cell counts mirrored that seen in the total WBC counts. Little enhancement in kill of lymphocytes (compared to CCNU alone) was observed when MISO was added to the CCNU. These findings were similar to previous reports on nitrosourea effects on WBC numbers in mice (Anderson *et al.*, 1975). So Day 3, which showed a nadir for the single-dose CCNU treatments as well as for the CCNU–MISO combination, was chosen for subsequent WBC toxicity evaluations.

Fig. 4 shows total WBC counts 3 days after treatment with a range of CCNU doses given alone or simultaneously with either 1·0 or 0·5 mg/g MISO. The data indicate that, unlike the findings in the previously described preliminary study, when complete dose–response curves for CCNU and CCNU+MISO were obtained,

FIG. 4.—Total WBC counts as a function of the CCNU dose measured 3 days after treatment with CCNU alone (solid symbols) or CCNU + 0·5 mg/g MISO (harlequin symbols) or CCNU + 1·0 mg/g MISO (open symbols). Different shapes represent different experiments. Each data point is the mean ± s.d. of a sample of 5 mice.

TABLE II.—*The enhancing effect of MISO on the cytotoxicity of CCNU in tumour and normal tissues*

| Tumour models | Endpoints | MISO dose (mg/g) | approx. ER |
|---|---|---|---|
| KHT sarcoma | Clonogenic cell survival | 0·25 | 1·9* |
| | | 0·5 | 2·1* |
| | | 1·0 | 2·4* |
| | Tumour growth delay | 0·25 | 1·3 |
| | | 0·5 | 1·7 |
| | | 1·0 | 1·9 |
| MT-1 mammary tumour | Tumour growth delay | 0·5 | 1·5 |
| RIF-1 tumour | Tumour growth delay | 1·0 | 2·0 |
| Normal tissues | | | |
| Gut | $LD_{50/7}$ | 0·5 | 1·2* |
| | | 1·0 | 1·4* |
| Marrow | Peripheral WBC | 0·5 | 1·0 |
| | | 1·0 | 1·0 |

* Siemann (1981).

there was no consistent enhancement of CCNU-induced WBC toxicity by MISO. This was especially so for CCNU + 0·5 mg/g MISO (right-hand panel).

Table II summarizes the results of combination therapy with CCNU and MISO for tumour responses and normal-tissue toxicities in the present study, as well as in a previous report (Siemann, 1981). The data indicate less enhancements of CCNU toxicity by addition of MISO for endpoints of peripheral WBC numbers than for gut lethality. It should be noted, however, that, in mice, gut toxicity is probably the more relevant factor defining single-dose nitrosourea toxicity (Blackett *et al.*, 1975). The tumour-response modification by the CCNU–MISO combination in general was larger than the modification in the normal tissue. This enhancement appears to be very similar in the 3 tumour models studied.

### DISCUSSION

Combinations of the nitroimidazole radiation sensitizer MISO and conventional chemotherapeutic agents such as cyclophosphamide (CY), BCNU, CCNU and melphalan (L-PAM) have been shown to enhance tumour cytotoxicity *in vivo* (Clement *et al.*, 1980; Rose *et al.*, 1980; Tannock, 1980*b*; Mulcahy *et al.*, 1981; Siemann, 1981; Law *et al.*, 1981; Twentyman, 1981). As has been reported previously (Siemann, 1981), the combination of CCNU with MISO was particularly effective in the KHT sarcoma. The investigation of this combination has been extended in the present study, and tumour growth-delay dose–response curves for CCNU doses combined with a range of MISO doses were obtained (Fig. 1). The results indicated that MISO could enhance the response of the KHT sarcoma to single doses of CCNU, even when the MISO dose was as low as 0·25 mg/g. This observation was qualitatively similar to the previous finding in the KHT sarcoma using a clonogenic-cell survival assay (Siemann, 1981). However, in that investigation larger ERs were obtained than in the present report using tumour-growth delay as the endpoint (Table II). Larger ERs for BCNU + MISO have also been observed in our laboratories when tumour response was assessed by *in vivo* to *in vitro* clonogenic-cell survival than using *in situ* tumour growth delay (Mulcahy *et al.*, submitted). Such apparent discrepancies between these two assays are not uncommon, and have previously been observed by many other authors studying the effects of a variety of treatments including radiation, chemotherapy, or combined-modality therapies on tumour response (Stephens & Peacock, 1977; Begg *et al.*, 1980; McNally & de Ronde, 1980; Twentyman, 1980; Hill, 1980).

In order to determine whether the enhanced tumour responses to CCNU–MISO combinations were specific to the KHT sarcoma, the effect of such treatments also were evaluated in the RIF-1 tumour as well as in a first-generation transplanted mammary tumour. Even though CCNU alone was found to be less effective in these two systems than in the KHT tumour, the relative ERs on the addition of MISO were similar (Fig. 2 and Table II).

Since other nitroimidazole sensitizers have been used or considered for clinical application, their ability to enhance to toxicity of CCNU also was tested. For comparison, these sensitizers were combined with CCNU at the same mmol/kg dose as MISO. Complete dose–response curves were not determined, yet even the single-dose combinations (Table I) indicated that, when administered simultaneously with CCNU, MISO was clearly superior to METRO, Ro-05-9963, SR-2508 and SR-2555 in enhancing the efficacy of CCNU in the KHT sarcoma. However, the data in Fig. 3 showed that in the RIF-1 tumour, SR-2508 also enhanced the effect of CCNU, though only for large CCNU doses. These results for different nitroimidazole sensitizers clearly require further investigation.

As we have suggested previously (Mulcahy *et al.*, 1981; Siemann, 1981), the enhancement of tumour response to alkylating chemotherapeutic agents by nitroimidazole sensitizers could be due to: (1) cytotoxicity of sensitizer in cells spared by the anti-tumour agents, (2) altered drug pharmacokinetics, (3) sensitization of the tumour to the chemotherapeutic agent by the sensitizer, and (4) inhibition of the repair of the chemotherapeutic agent's potentially lethal damage (PLD) by the sensitizer.

The first possibility appears highly unlikely, since MISO causes no tumour-growth delay and minimal cell kill in the KHT sarcoma until doses in excess of 1 mg/g are administered (Mulcahy *et al.*, 1981*a*, submitted; Siemann, 1981). Yet even doses as low as 0·25 and 0·5 mg/g of this sensitizer can enhance considerably the tumour response to CCNU (Table II).

With respect to the altered-pharmacokinetics hypothesis, results to date are conflicting. Findings in our laboratory with a related nitrosourea (BCNU) and CY have indicated that the MISO pharmacokinetics are not altered when these two agents are combined with this sensitizer. Also, the addition of MISO has been shown not to alter the pharmacokinetics of BCNU in mice (Tannock, 1980*b*) or in patients (Urtasun *et al.*, 1982). Similar studies on the *in vivo* pharmacokinetics of CCNU in the presence or absence of MISO do not exist. However, since CCNU, unlike BCNU, undergoes ring hydroxylation by liver microsomal enzymes (May *et al.*, 1974; Hilton & Walker, 1975; Wheeler *et al.*, 1977) the possibility arises that the enhanced tumour response on the addition of MISO to CCNU treatment could be the result of altered CCNU metabolism in the presence of the sensitizer. The observations in the KHT sarcoma could be interpreted as supporting this view, since MISO, which undergoes oxidative demethylation, probably by liver microsomal mixed-function oxidases, enhances the cytotoxicity of CCNU, whereas Ro-05–9963 and SR-2508 are not metabolized in this manner (White *et al.*, 1980) and are relatively ineffective (Table I). However, other have shown that hydroxylation of the cyclohexane ring of CCNU leads to metabolites which have slightly better therapeutic indices (Wheeler *et al.*, 1977) or tumour efficacies similar (Johnston *et al.*, 1975) to that of the parental CCNU. In addition, while an enhanced rate of CCNU metabolism has been reported in phenobarbital-pretreated animals (Hilton & Walker, 1975) such treatments did not alter significantly CCNU anti-tumour activity (Levin *et al.*, 1979). However, preliminary data in this laboratory (Siemann, unpublished) have indicated that phenobarbital pretreatment can reduce, by about the same factor, both the response of the KHT sarcoma and the degree of normal-tissue toxicity due to CCNU administration. Also, Workman & Twentyman (1982), have shown that SKF-525A, an inhibitor of drug-metabolizing enzymes, can enhance the tumoricidal effects of CCNU. Finally, in contrast to the results obtained with MISO, the 5-nitroimidazole METRO, which undergoes oxidative metabolism (Ings *et al.*, 1966), did not enhance the response of the KHT sarcoma to CCNU. In addition, SR-2508 effectively enhanced the efficacy of CCNU in the RIF-1 tumour (Fig. 3), though only at the largest doses of CCNU. Thus, whilst altered chemotherapeutic-agent metabolism may not be the primary mechanism for the enhanced tumour response to CCNU-sensitizer combinations, further evaluation of the interactions between anti-tumour agents and sensitizers is clearly required.

Sensitization of the tumour cells to the chemotherapeutic agent, perhaps analogous to sensitization to radiation of hypoxic cells, is another possible mechanism of the sensitizer effect. In support of this view, Tannock (1980*b*) has shown that, whilst the serum of mice treated with BCNU or BCNU + MISO was equally toxic to cells exposed *in vitro* under aerobic conditions, the combination was much more toxic to anaerobic cells. The present results yield little information about this possible mechanism. However, other studies (Siemann & Mulcahy, 1982) have indicated that, depending on the chemotherapeutic agent used, the enhanced tumour response to MISO–chemotherapeutic-agent combinations may result both from a potentiation of the chemotherapeutic agent's cytotoxic effects and/or an inhibition of the clonogenic-cell recovery, measured as a function of time after treatment. When CCNU and MISO were combined, the major effect of the sensitizer appeared to be on the recovery in clonogenic-cell survival, such that 24 h after treatment tumour-cell survival in mice receiving the combination treatment was about 2% of that in mice given only CCNU. Similar cell-survival responses have also been observed by Law *et al.* (1981) for combinations of CY and MISO in the RIF-1 tumour. Thus, although the present investigation does not allow us to determine the mechanism of the enhanced tumour response with certainty, other data suggest that inhibition of recovery of clonogenic-cell survival plays a major role in the large ERs observed with the CCNU–MISO combination in the KHT sarcoma.

It is of interest to note that, unlike MISO, the other sensitizers evaluated in combination with 20 mg/kg CCNU were ineffective in the KHT sarcoma (Table I). At an equimolar dose, SR-2508 did enhance the response of the RIF-1 tumour to CCNU, though not to the same extent as MISO, and not at all at lower doses of CCNU (Fig. 3). Enhancement of CY damage by SR-2508 has also been found in the RIF-1 tumour (Law *et al.*, 1981) and KHT sarcoma (Siemann & Sutherland, 1982). These findings imply that there may be some degree of both tumour and chemotherapeutic-agent specificity when different radiosensitizers are used to modify the tumoricidal action of anti-tumour agents.

Experiments in our laboratories with nitrosoureas of different alkylating and carbamoylating activities, have also shown that the particular nitrosourea chosen may be a key factor in the level of enhancement of tumour-cell kill on the addition of MISO. Of the nitrosoureas studied, CCNU, which was the most carbamoylating agent tested, gave the largest enhancement ratio when combined with MISO. In contrast, the addition of MISO to chlorozotocin, a nitrosourea with relatively little carbamoylating activity, had no effect (Mulcahy *et al.*, submitted). Further evaluations of various nitrosoureas and radiation sensitizers *in vitro* are in progress.

Finally the question of enhanced therapeutic gain needs to be considered. Using gut toxicity and depression of WBC numbers as endpoints, investigations in this laboratory have indicated a greater effect in the tumour than the normal tissues when CCNU and MISO are combined. These findings imply that an improved therapeutic result may be achieved through such a combination. Nevertheless, the extension of combinations of chemotherapeutic agents and radiation sensitizers to the clinic should be done cautiously.

The investigations reported in this paper were supported by NIH Grants CA-11051, CA-20329 and CA-11198. Excellent technical assistance was provided by J. Beilman. This work was presented in part at the 29th Annual Meeting of the Radiation Research Society, June 1981, Minneapolis, Minnesota.

### REFERENCES

Anderson, T., McMenamin, M. & Schein, P. S. (1975) Chlorozotoxin, 2-[3-(2-chlorethyl)-3-nitrosoureido]-D-glucopyranose, an antitumor agent with modified bone marrow toxicity. *Cancer Res.*, **35**, 761.

Begg, A. C., Fu, K. K., Kane, L. J. & Phillips, T. L. (1980) Single-agent chemotherapy of a solid murine tumor assayed by growth delay and cell survival. *Cancer Res.*, **40**, 145.

Blackett, N. M., Courtenay, V. D. & Mayer, S. M. (1975) Differential sensitivity of colony-forming cells of hemopoietic tissue, Lewis lung carcinoma, and B16 melanoma to three nitrosoureas. *Cancer Chemother. Rev.*, **59**, 929.

Clement, J. J., Gormann, M. S., Wodinsky, I., Catane, R. & Johnson, R. K. (1980) Enhancement of antitumor activity of alkylating agents by the radiation sensitizer misonidazole. *Cancer Res.*, **40**, 4165.

Hill, R. P. (1979) Combined nitrogen mustard-radiation studies with a mouse tumor. *Int. J. Radiat. Oncol. Biol. Phys.*, **5**, 1611.

Hill, R. P. (1980) Radiation-induced changes in the *in vivo* growth rate of KHT sarcoma cells: Implications for the comparison of growth delay and cell survival. *Radiat Res.*, **83**, 99.

Hill, R. P. & Stanley, J. A. (1975) The response of hypoxic B16 melanoma cells to *in vivo* treatment with chemotherapeutic agents. *Cancer Res.*, **35**, 1147.

Hilton, J. & Walker, M. D. (1975) Hydroxylation of 1-(2-chloroethyl)-3-cyclohexyl-1-nitrosourea. *Biochem. Pharmacol.*, **24**, 2153.

Ings, R. M. J., Law, G. L. & Parnell, E. W. (1966) The metabolism of metronidazole (1-2′-hydroxyethyl-2-methyl-5-nitroimidazole). *Biochem. Pharmacol.*, **15**, 515.

Johnston, T. P., McCaleb, G. S. & Montgomery, J. A. (1975) Synthesis and biologic evaluations of major metabolites of N-(2-chloroethyl)-N′-cyclohexyl-N-nitrosourea. *J. Med. Chem.*, **18**, 634.

Kallman, R. F., Silini, J. & van Putten, L. M. (1967) Factors influencing the quantitation of the *in vivo* survival of cells from solid tumors. *J. Natl Cancer Inst.*, **39**, 539.

Law, M. P., Hirst, D. G. & Brown, J. M. (1981) The enhancing effect of misonidazole on the response of the RIF-1 tumour to cyclophosphamide. *Br. J. Cancer*, **44**, 208.

Levin, V. A., Stearns, J., Byrd, A., Finn, A. & Weinkam, J. (1979) The effect of phenobarbital pretreatment on the antitumor activity of 1,3-bis-(2-chloroethyl)-1-nitrosourea [BCNU], 1-(2-chloroethyl)-3-cyclohexyl-1-nitrosourea [CCNU] and 1-(2-chloroethyl)-3-(2,6-dioxo-3-piperyl-1-nitrosourea [PCNU), and on the pharmapharmacokinetics and biotransformation of BCNU. *J. Pharmacol. Exp. Therap.*, **208**, 1.

May, H. E., Boose, R. & Reid, D. J. (1974) Hydroxylation of the carcinostatic 1-(2-chloroethyl)-3-cyclohexyl-1-nitrosourea by rat liver microsomes. *Biochem. Biophys. Res. Commun.*, **57**, 426.

McNally, N. J. & de Ronde, J. (1980) Radiobiological studies of tumours *in situ* compared to cell survival. *Br. J. Cancer*, **41** (Suppl. IV), 259.

Mulcahy, R. T., Siemann, D. W. & Sutherland, R. M. (1981) *In vivo* response of KHT sarcomas to combination chemotherapy with radiosensitizers and BCNU. *Br. J. Cancer*, **43**, 93.

Mulcahy, R. T., Siemann, D. W. & Sutherland, R. M. Nitrosourea–misonidazole combination chemotherapy: Effect on KHT sarcomas, bone marrow stem cells and gut. *Br. J. Cancer* (submitted).

Noether, J. (1971) *Introduction to Statistics—A Fresh Approach.* Boston: Houghton Mifflin.

Rose, C. M., Millar, J. L., Peacock, J. H., Phelps, T. A. & Stephens, T. C. (1980) *Differential Enhancement of Melphalan Cytotoxicity in Tumour Normal Tissue by Misonidazole.* New York: Masson Publishers. p. 10.

Siemann, D. W. (1981) The *in vivo* combination of the nitroimidazole misonidazole and the chemotherapeutic agent CCNU. *Br. J. Cancer*, **43**, 367.

Siemann, D. W. & Mulcahy, R. T. (1982) Cell survival recovery kinetics in the KHT sarcoma following treatment with five alkylating agents and misonidazole. *Int. J. Radiat. Oncol. Biol. Phys.* (in press).

Siemann, D. W., Hill, R. P. & Bush, R. S. (1977) The importance of pre-irradiation breathing times of oxygen and carbogen (5% $CO_2$: 95% $O_2$) on the *in vivo* radiation response of a murine sarcoma. *Int. J. Radiat. Oncol. Biol., Phys.*, **2**, 903.

Siemann, D. W. & Sutherland, R. M. (1980) *In vivo* tumor response to single and multiple exposures of adriamycin. *Eur. J. Cancer*, **16**, 1433.

Siemann, D. W. & Sutherland, R. M. (1982) Combinations of cyclophosphamide and misonidazole in the KHT sarcoma. *Int. J. Radiat. Oncol. Biol. Phys.* (in press).

Stephens, T. C. & Peacock, J. H. (1977) Tumour volume response, initial cell kill and cellular repopulation in B16 melanoma treated with cyclophosphamide and 1-(2-chloroethyl)-3-cyclohexyl-1-nitrosourea. *Br. J. Cancer*, **36**, 313.

Sutherland, R. M., Siemann, D. W. & Eddy, H. A. (1978) Influence of mode of growth of EMT6 tumour cells on response to Adriamycin. *Radiat. Res.*, **74**, 578.

Sutherland, R. M., Eddy, H. A., Bareham, B., Reich, K. & Vanantwerp, D. (1979) Resistance to adriamycin in multicellular spheroids. *Int. J. Radiat. Oncol. Biol. Phys.*, **5**, 1225.

Sutherland, R. M., Bareham, B. J. & Reich, K. A. (1980) Cytotoxicity of hypoxic cell sensitizers in multicell spheroids. *Cancer Clin. Trials*, **3**, 73.

Tannock, I. (1980*a*) *In vivo* interaction of anticancer drugs with misonidazole or metronidazole: Methotrexate, 5-fluorouracil and adriamycin. *Br. J. Cancer*, **42**, 861.

Tannock, I. (1980*b*) *In vivo* interaction of anticancer drugs with misonidazole or metronidazole: Cyclophosphamide and BCNU. *Br. J. Cancer*, **42**, 871.

Thomson, J. E. & Rauth, A. M. (1974) An *in vitro* assay to measure the viability of KHT tumor cells not previously exposed to culture conditions. *Radiat. Res.*, **58**, 262.

Twentyman, P. R. (1980) Experimental chemotherapy studies: Intercomparison of assays. *Br. J. Cancer*, **41** (Suppl. IV), 279.

Twentyman, P. R. (1981) Modification of tumour and host response to cyclophosphamide by misonidazole and WR-2721. *Br. J. Cancer*, **43**, 745.

Twentyman, P. R., Brown, J. M., Gray, J. W., Franko, A. J., Scoles, M. A. & Kallman, R. F. (1980) A new mouse tumor model system (RIF-1) for comparison end-point studies. *J. Natl Cancer Inst.*, **64**, 595.

Twentyman, P. R. & Workman, P. (1982) The effect of radiosensitizer pretreatment on the response of the RIF-1 mouse sarcoma to cytotoxic drugs. *Int. J. Radiat. Oncol. Biol. Phys.* (in press).

Wheeler, G. P., Johnson, T. P., Bowden, B. J., McCaleb, G. S., Hill, D. L. & Montgomery, J. A. (1977) Comparison of the properties of metabolites of CCNU. *Biochem. Pharmacol.*, **26**, 2331.

White, R. A., Workman, P. & Brown, J. M. (1980) The pharmacokinetics, tumor and neural tissue penetrating properties in the dog of SR-2508 and SR-2555—hydrophylic radiosensitizers potentially less toxic than misonidazole. *Radiat. Res.*, **84**, 542.

Workman, P. & Twentyman, P. R. (1982) Enhancement by electron-affinic agents of the therapeutic effects of cytotoxic agents against the KHT tumour: Structure–activity relationships. *Int. J. Radiat. Oncol. Biol. Phys.* (in press).

Urtasun, R. C., Tanasiuk, H., Fulton, D., Raleigh, J., Rabin, H. R., Turner, R. & Agboola, P. (1982) Pharmacokinetic interaction of BCNU and misonidazole in humans. *Int. J. Radiat. Oncol. Biol. Phys.* (in press).